# *CARTPT* and FSH Signaling in Ovarian Follicle Development as an Emerging Link to Oocyte Quality and Female Infertility

**DOI:** 10.3390/cimb48101024

**Published:** 2026-10-03

**Authors:** Charalampos Voros, Fotios Chatzinikolaou, Georgios Papadimas, Diamantis Athanasiou, Antonia Athanasiou, Aikaterini Athanasiou, Nikolaos Thomakos, Panagiotis Antsaklis, Dimitrios Loutradis, Georgios Daskalakis

**Affiliations:** 1 Department of Obstetrics and Gynecology, ‘Alexandra’ General Hospital, National and Kapodistrian University of Athens, 80 Vasilissis Sofias Avenue, 11528 Athens, Greece; 2 Laboratory of Forensic Medicine and Toxicology, School of Medicine, Aristotle University of Thessaloniki, 54124 Athens, Greece; 3 Athens Medical School, National and Kapodistrian University of Athens, 15772 Athens, Greeceloutradi@otenet.gr (D.L.); 4 IVF Athens Reproduction Center, 15123 Maroussi, Greece

**Keywords:** *CARTPT*, FSH signaling, granulosa cells, leptin, oocyte quality, female infertility

## Abstract

Not all ovarian follicles respond to follicle-stimulating hormone (FSH) in the same way. This difference is usually attributed to FSH receptor expression and local ovarian factors, but the role of *CARTPT* has received far less attention. Studies in granulosa cells have shown that cocaine- and amphetamine-regulated transcript (CART) can reduce FSH-induced cAMP production, aromatase expression, and estradiol synthesis. *CARTPT* expression has also been found to be higher in subordinate and atretic follicles in several animal models. Its regulation by leptin suggests a possible connection between metabolic status and follicular function. Early human findings have linked follicular CART expression with ovarian response and oocyte yield. In cattle and buffalo, CART suppressed FSH-stimulated cAMP production, aromatase expression, and estradiol output in granulosa cells and marked follicles destined for atresia, a pattern not confirmed in the one available porcine study. The receptor mediating these effects in the ovary remains unidentified, though a CART receptor was recently confirmed in another endocrine tissue. Human evidence linking *CARTPT* to ovarian response and oocyte quality remains preliminary and correlative. *CARTPT* therefore emerges as a mechanistically well-supported but clinically unproven contributor to FSH resistance and impaired oocyte quality, and this review outlines the loss-of-function and prospective human studies needed to establish its clinical relevance.

## 1. Introduction

Follicular development in the mammalian ovary is not a uniform process. Within a single growing cohort, only a small number of follicles reach the preovulatory stage and acquire full estrogen-producing capacity; the rest undergo atresia at various points along the way [1]. In monovular and monotocous species alike, this selection is tightly coupled to the follicle’s ability to respond to follicle-stimulating hormone (FSH), and the loss of that responsiveness typically precedes any morphological sign of degeneration. For decades, the explanation offered for this divergence has centered on two factors: differences in FSH receptor density on granulosa cells, and the local paracrine environment created by growth factors, cytokines, and steroid feedback within the follicle itself [1]. They describe how follicles differ in their capacity to receive and interpret the FSH signal, without fully accounting for what actively suppresses that signal in follicles destined for atresia.

One candidate that has received comparatively little attention in this context is cocaine- and amphetamine-regulated transcript (CART), the peptide product of the *CARTPT* gene [2,3]. CART was originally identified in the rat striatum as a transcript upregulated by psychostimulant exposure, and its physiological role only became clear once it was shown to function as a hypothalamic satiety factor whose expression is induced by leptin [4]. In the years that followed, CART was characterized extensively in the central nervous system, where it participates in feeding behavior, stress responses, and reward circuitry, and its actions were later extended to peripheral tissues including the pancreas and gastrointestinal tract. None of this pointed toward a role in the ovary, and indeed, when CART was first detected there, the finding came somewhat by accident, during the construction of a bovine oocyte cDNA library rather than through any hypothesis-driven search for local ovarian regulators [5].

That initial observation turned out to be consequential. CART protein and mRNA were localized to granulosa and thecal cells of large antral follicles in cattle, and their abundance tracked inversely with follicle health: *CARTPT* expression was higher in subordinate and atretic follicles than in the dominant follicle of a follicular wave [6]. When granulosa cells were exposed to the mature CART peptide in vitro, the FSH-stimulated rise in cAMP was blunted, aromatase mRNA expression fell, and estradiol synthesis declined accordingly [7,8]. Later work traced this inhibition to a more specific point in the signaling cascade, showing that CART shortens the duration of FSH-induced ERK1/2 and Akt activation by altering the expression and turnover of specific MAPK phosphatases, and that the granulosa cell’s responsiveness to CART itself changes across the stages of a follicular wave [9,10]. Taken together, this line of work, carried out largely by one research group over roughly a decade, builds a coherent picture in cattle: CART functions as a locally produced signal that dampens the follicle’s response to FSH, and its expression pattern places it squarely among the candidates that could help explain why some follicles fail to keep pace with the dominant one.

Whether this mechanism operates only in cattle, or reflects something closer to a conserved feature of follicular biology, is less settled. CART has since been described in porcine and avian ovaries as well as in buffalo, with broadly similar associations between CART abundance and follicle status, though the details of localization differ somewhat between species [11]. Notably, CART is reportedly absent from the mouse ovary altogether, which complicates any assumption that findings from one species will translate directly to another and raises a genuine question about how representative the bovine model is of ovarian CART biology more broadly.

A second strand of evidence connects this local ovarian mechanism back to the peptide’s original identity as a leptin-regulated satiety factor. If leptin induces CART in the hypothalamus, an analogous relationship in the ovary would offer a mechanistic bridge between systemic metabolic status and follicular outcome, distinct from leptin’s better-known central effects on the hypothalamic–pituitary–ovarian axis. This possibility was tested directly in mice, where diet-induced obesity and the associated hyperleptinemia were shown to increase granulosa cell CART expression, with downstream effects on cAMP signaling, aromatase expression, and ovulation rate that mirrored the effects of CART administration alone [12]. The same study extended the observation to a small human cohort undergoing in vitro fertilization, reporting a positive correlation between body mass index, granulosa cell *CARTPT* mRNA, and CART peptide concentration in follicular fluid. A considerably larger human cohort has since linked follicular fluid CART directly to ovarian response and oocyte quality [13]; both should be read with the caution that correlative clinical studies warrant.

The broader human literature on obesity, leptin, and oocyte quality does little to simplify the picture. Follicular fluid leptin concentrations have been reported to correlate positively with fertilization rates in some cohorts, negatively with embryo quality in others, and not at all with reproductive outcomes in still others, a pattern that likely reflects differences in patient selection, assay methods, and the confounding influence of body composition rather than any single consistent biological relationship. It is against this inconsistent background that a more specific, receptor-level mechanism such as CART becomes worth examining, not as a replacement for the leptin literature but as a possible piece of the pathway connecting adiposity to impaired follicular function. Complicating this further, the identity of the CART receptor itself remains contested: a G protein-coupled receptor, GPR160, has been proposed as a candidate, but subsequent binding studies have failed to confirm this assignment, leaving the downstream signaling architecture of CART only partly resolved even outside the reproductive context.

Our review draws together the available evidence on *CARTPT* in ovarian follicular development, with particular attention to its interaction with FSH signaling in granulosa cells, its proposed role in follicle selection and atresia across the species in which it has been studied, and the limited but suggestive data linking it to oocyte quality and infertility in women. Where the evidence is preclinical, this is stated explicitly; where human data exist, their scope and limitations are described rather than extrapolated beyond what the underlying studies support. The aim is not to argue that *CARTPT* is an established clinical biomarker, but to lay out clearly what is known, what remains speculative, and where the most useful next steps in this line of research are likely to lie.

## 2. Material and Methods

Because this article is a narrative rather than a systematic review, no protocol was registered in advance, and no attempt was made to quantify the number of records identified, screened, or excluded at each stage. What follows is intended as a transparent account of how the literature was located and selected, not as a claim to the kind of reproducible, auditable search process that a systematic review would require.

The primary source was PubMed, searched most recently on 15 January 2026. Search terms were built around three overlapping concept groups, combined with Boolean operators. The first group covered the peptide itself: “CARTPT,” “cocaine- and amphetamine-regulated transcript,” and “CART,” used both as free-text terms and, where indexed, under the MeSH heading *Nerve Tissue Proteins* with the subheading restricted to reproductive contexts where possible. The second group covered the ovarian and endocrine setting: “granulosa cell,” “follicle-stimulating hormone,” “FSH receptor,” “follicular atresia,” “dominant follicle,” “ovarian follicle,” and “aromatase,” several of which map to the MeSH terms *Granulosa Cells*, *Follicle Stimulating Hormone*, and *Ovarian Follicle*. The third group addressed the metabolic and clinical dimension: “leptin,” “obesity,” “oocyte quality,” “follicular fluid,” and “in vitro fertilization,” corresponding to MeSH terms including *Leptin*, *Obesity*, *Oocytes*, and *Fertilization* in Vitro. A representative combined search string took the form (CARTPT OR “cocaine- and amphetamine-regulated transcript”) AND (granulosa OR ovary OR follicle) AND (FSH OR leptin OR oocyte), with narrower variants run separately for each concept pair to avoid missing papers that used only one term from each group.

No fixed lower date limit was applied. This was a deliberate choice rather than an oversight: the foundational paper linking CART to leptin dates to 1998, and the core experimental work establishing CART as an intraovarian regulator spans roughly 2004 to 2013, concentrated within a small number of research groups working primarily in cattle. Imposing a conventional five- or ten-year window, as is often reasonable for faster-moving clinical fields, would have excluded the papers that this review depends on most heavily. Searches were, however, run through to 15 January 2026 to capture more recent extensions of the model into other species and into human follicular fluid.

Beyond the direct database search, citation tracking was used extensively. Because the *CARTPT*-ovary literature is small and tightly interconnected, following the reference lists of the earliest papers proved more productive in places than repeated database queries, and several relevant studies particularly those in porcine, avian, and buffalo models were located this way rather than through the original search strings. Where a paper cited a prior study from the same or a closely related group, that prior study was retrieved and checked directly rather than relied upon secondhand.

Searches were restricted to English-language publications and to a single database, PubMed. Supplementing this with Scopus, Web of Science, or Embase was not undertaken here, so relevant work indexed only in one of those databases may have been missed; this risk is likely reduced, though not eliminated, by the small size and tight interconnection of the *CARTPT*-ovary literature and by the citation-tracking step described above. This single-database, English-only strategy is accordingly treated as a limitation of the present review rather than a settled methodological choice. Both primary experimental studies and existing narrative or systematic reviews were consulted, but reviews were used only for orientation, background framing, and identification of additional primary sources; no claim made in the results or discussion of the present review rests on a review article alone without tracing it back to the underlying primary study. Preference in study selection was given, in descending order, to: studies directly measuring *CARTPT* mRNA or CART peptide in ovarian tissue, granulosa cells, thecal cells, or follicular fluid; mechanistic in vitro studies examining CART effects on FSH-stimulated signaling (cAMP, ERK1/2, Akt, aromatase expression, estradiol production); studies establishing the CART-leptin relationship, whether in central or peripheral tissue; and human clinical studies reporting follicular fluid leptin or CART in relation to oocyte or embryo outcomes.

Exclusion criteria were kept deliberately narrow, consistent with the exploratory nature of a narrative review on an understudied topic. Studies were excluded only if CART was addressed exclusively in a central nervous system or non-reproductive peripheral context with no stated or plausible link to ovarian function, or if a paper’s relevance to the ovarian mechanism could not be established from its own content, for example, where CART was mentioned only in passing within a broader review of neuropeptide biology. Species was not used as an exclusion criterion, given how few species have been studied at all; findings from cattle, pigs, poultry, buffalo, mice, and humans are all included but are explicitly labeled by species throughout the results, so that evidence of differing strength and directness is never presented as though it were equivalent.

For the section addressing leptin and human IVF outcomes, which functions as clinical context surrounding the direct human *CARTPT* studies rather than as *CARTPT* evidence itself, the search was intentionally broader but the selection more conservative: priority was given to studies that measured follicular fluid leptin concentrations alongside a defined, quantifiable reproductive outcome, such as fertilization rate, embryo quality score, oocyte maturity, or pregnancy outcome, in a stated patient population, rather than studies reporting leptin as one variable among many without a clear outcome linkage.

## 3. CART Biology: From the Hypothalamus to the Periphery

Before turning to the ovary, it is worth setting out what is actually established about CART as a molecule: its gene, its processing, and its signaling behavior, since much of what makes an ovarian role plausible rests on properties first worked out in an entirely different tissue.

### 3.1. Gene, Prohormone Processing, and Discovery

CART entered the literature not through a planned search for a reproductive or metabolic regulator, but as an incidental finding in psychopharmacology. Using PCR differential display to screen for rat brain transcripts responsive to acute psychostimulant exposure, Douglass and colleagues identified a previously uncharacterized striatal mRNA whose abundance rose four- to fivefold within hours of cocaine or amphetamine administration [13]. The gene, later designated *CARTPT*, was named for the pharmacological circumstance of its discovery rather than for any known physiological role, and for several years that is essentially all it was: a drug-responsive transcript of unclear biological significance [14].

Like most neuropeptide genes, *CARTPT* does not encode a mature, active peptide directly. It encodes a prepro-hormone that must be proteolytically processed before it becomes biologically active [15]. Thim and colleagues purified and sequenced native CART peptides from rat adrenal gland, hypothalamus, nucleus accumbens, and pituitary tissue, and showed that processing of the precursor is tissue-specific, generating a family of related fragments rather than a single uniform product [16]. In the rat, the gene gives rise to a 116-amino-acid prohormone, or a 129-residue variant produced by alternative splicing that inserts an additional 13 residues into the coding sequence; only the shorter form has a clear human equivalent. Cleavage occurs at mono- and dibasic sites within the precursor, consistent with processing by the prohormone convertases PC1/3 and PC2, the same convertase pair responsible for maturation of proopiomelanocortin and proinsulin. The predominant bioactive fragment recovered across tissues corresponds to the C-terminal region of the precursor, a peptide of roughly 48 residues stabilized by three intramolecular disulfide bonds. This disulfide-constrained structure is not a minor biochemical detail: it is what gives the mature CART peptide resistance to proteolytic degradation once secreted, allowing it to persist as a stable extracellular signal rather than being rapidly broken down, a property that becomes directly relevant later in this review, when CART peptide is measured in follicular fluid rather than only within tissue extracts.

For roughly three years after its initial description, CART remained a curiosity of drug-response biology. Its transformation into a peptide of broader physiological interest came with the demonstration that it functions as a hypothalamic satiety factor whose expression is regulated by leptin. Kristensen and colleagues showed that CART mRNA in the arcuate nucleus falls sharply during food deprivation and is nearly undetectable in animal models of obesity with disrupted leptin signaling, while peripheral leptin administration to obese mice restores arcuate CART expression [4]. Centrally administered CART peptide, in turn, suppresses both normal and starvation-induced feeding and blocks the orexigenic action of neuropeptide Y [4]. This paper did more than add a new satiety peptide to the list. It placed CART downstream of leptin within the arcuate melanocortin circuitry, as one of the mechanisms through which a peripheral adiposity signal is translated into a central behavioral output. That specific relationship, leptin transcriptionally driving CART expression with CART then producing an effect distinct from any direct action of leptin itself, is the template later invoked, with substantial modification, to explain a proposed mechanism in the ovary, discussed in Section 7. It is worth treating this as an analogy rather than an established equivalence: a shared upstream signal in two different tissues does not by itself imply a shared downstream signaling architecture, particularly given how much about CART’s own receptor pharmacology remains unsettled, as described below.

Beyond the arcuate nucleus, subsequent immunohistochemical and in situ hybridization work mapped CART expression across the paraventricular and dorsomedial hypothalamic nuclei, the lateral hypothalamic area, and projections extending into the nucleus accumbens, ventral tegmental area, and spinal cord, a distribution consistent with CART’s involvement in reward processing and in the psychostimulant response from which the gene originally took its name [17]. CART expression is not restricted to the central nervous system. It has also been described in enteroendocrine cells of the gastrointestinal tract and in the endocrine pancreas, where it has been reported to modulate insulin and glucagon secretion from beta and alpha cells respectively [15]. This peripheral, cell-autonomous mode of action, a locally produced peptide acting on the secretory behavior of the very cell population that produces it or on its immediate neighbors, is arguably a closer precedent for the granulosa cell biology discussed in later chapters than the long-range hypothalamic circuitry with which CART is most commonly associated [18].

### 3.2. Receptor Signaling: An Unresolved Pharmacology

One aspect of CART biology has remained genuinely unsettled throughout this expanding body of work: the molecular identity of its receptor. For more than two decades after the peptide’s discovery, CART was studied entirely as a ligand in search of a receptor [19]. Radioligand binding studies using iodinated CART fragments identified specific, saturable, high-affinity binding sites in neuronal cell lines, including AtT20 pituitary corticotroph cells and PC12 pheochromocytoma cells, with binding that increased upon neuronal differentiation and was sensitive to guanine nucleotides, a pharmacological signature consistent with coupling to a heterotrimeric G protein rather than to an ion channel or enzyme-linked receptor [20]. Functional studies in these systems showed that CART engagement inhibits forskolin-stimulated adenylate cyclase activity and reduces intracellular cAMP accumulation, a pattern consistent with coupling to an inhibitory Gi/Go-type G protein [15]. This particular signature, Gi/Go-mediated suppression of cAMP production, reappears essentially unchanged in its basic pharmacology in the granulosa cell studies discussed in Chapter 5, which is one of the more internally consistent features of the CART literature even in the continued absence of a confirmed receptor.

A specific candidate, the orphan G protein-coupled receptor GPR160, was proposed as the CART receptor on the basis of studies in neuropathic pain and central appetite regulation, where GPR160 knockdown attenuated the anorexigenic and antinociceptive effects of exogenous CART peptide and CART was reported to co-immunoprecipitate with GPR160 in a cancer cell line [20]. This assignment did not hold up under direct pharmacological testing. Freitas-Lima and colleagues examined CART peptide binding in PC12 cells, a line already established as expressing genuine CART binding sites, and found no detectable GPR160 expression there at all. When they extended the analysis to a cell line with high endogenous GPR160 expression and to two GPR160-transfected lines selected for their negligible baseline expression, they found no specific CART binding attributable to GPR160 in any of them, despite confirming GPR160 protein expression by immunocytochemistry in the same cells [21]. Their conclusion was direct: GPR160 cannot account for CART’s binding and signaling actions, and the identity of the true CART receptor, or receptors, remains open.

A more direct molecular candidate has since emerged from a different endocrine tissue. Lindqvist and colleagues combined RNA sequencing, co-immunoprecipitation, radioligand binding, proximity ligation assays, patch-clamp electrophysiology, and siRNA knockdown to identify GPR162 as a genuine CART receptor in human and mouse pancreatic islets: GPR162 immunoreactivity was present in 82% ± 13% of human beta cells and was also detected in alpha cells, and siRNA knockdown of GPR162 reduced the CART–GPR162 interaction by approximately 90% [19]. The same study re-examined GPR160 in islet tissue directly and found no GPR160 immunoreactivity and no CART-GPR160 interaction there, a result that reinforces rather than contradicts the exclusion reported by Freitas-Lima and colleagues. GPR162 is therefore the first CART receptor identified with this degree of methodological convergence, but the identification is tissue-specific: it was established in pancreatic islet cells, and neither GPR162 expression nor CART binding to GPR162 has been examined in the ovary, granulosa cells, or any other reproductive tissue. Whether GPR162 also accounts for the CART binding sites already described in bovine granulosa cells is therefore an open, directly testable question rather than a settled one.

This is not a minor dispute confined to receptor pharmacology as a subfield. It means that every downstream signaling effect attributed to CART, including the suppression of cAMP in neuronal and pituitary cells and the modulation of insulin and glucagon secretion in the pancreas [22], rests on the same unresolved receptor question as the ovarian effects this review focuses on. The inhibition of FSH-stimulated cAMP, ERK1/2, and Akt signaling in granulosa cells has been characterized through second-messenger and kinase readouts, without a molecularly confirmed receptor anchoring the first step of the pathway [23]. The same ambiguity applies directly to the ovarian literature: specific, saturable CART binding sites have been demonstrated on bovine granulosa cells, and their density has been shown to change with hormonal treatment and with the stage of the follicular wave, but whether these binding sites correspond to GPR160, to a distinct ovarian receptor entirely, or to more than one binding population within the same cell, has not been examined [10]. This gap in the underlying pharmacology is worth keeping in view throughout the mechanistic chapters that follow: what is described there is a well-characterized signaling cascade sitting on top of a receptor that, outside the pancreas, has not yet been molecularly identified.

## 4. Discovery and Local Expression of *CARTPT* in the Ovary

### 4.1. Identification in the Bovine Ovary

*CARTPT’s* route into reproductive biology did not begin with a hypothesis about follicle selection or gonadotropin resistance. It began as a byproduct of a genomics resource-building project. In the early 2000s, a group at Michigan State University set out to characterize the transcriptome of the bovine oocyte, on the reasoning that oocyte-derived and oocyte-associated transcripts were poorly represented in existing gene expression databases relative to somatic tissues [24]. Yao and colleagues constructed a cDNA library from bovine oocytes and used it to build a microarray platform intended as a general resource for identifying genes relevant to follicular development and early embryogenesis. This was not, in itself, a study designed to find a new regulator of FSH signaling; it was infrastructure, meant to be mined for candidate genes in whatever direction the sequence data pointed [24].

One of the transcripts recovered from this screen, when sequenced and compared against existing databases, matched *CARTPT*, a gene with no prior association to ovarian tissue in any species and one whose only established physiological role at that point was as a leptin-regulated hypothalamic satiety factor [4]. Kobayashi and colleagues followed up on this unexpected match with a dedicated study, using reverse transcription PCR, in situ hybridization, and immunohistochemistry to ask two separate questions: first, whether *CARTPT* transcript detected in a pooled oocyte-associated cDNA library actually reflected expression within the follicle at the tissue level, and second, if so, in which specific cellular compartment [5]. Both mRNA and immunoreactive CART peptide were detected in the granulosa cell layer of antral follicles, with weaker but consistent signal also present in the theca interna; expression was essentially undetectable in primordial and small preantral follicles, restricting *CARTPT’s* presence to the developmental window in which follicles have already become responsive to gonadotropin stimulation [25].

The experimental design that followed this localization work is what gives the 2004 paper its lasting influence on the field, more so than the initial expression screen itself. Rather than treating *CARTPT* as an isolated finding, Kobayashi and colleagues placed it within the established bovine model of follicular wave dynamics, in which a cohort of similarly sized antral follicles emerges together, one follicle is selected to become dominant and continues growing toward ovulation, and the remaining subordinate follicles cease growth and enter atresia within days of the deviation point [5]. Granulosa cells and follicular fluid were sampled from follicles at defined stages relative to this deviation from the largest, presumptive dominant follicle and from smaller, subordinate follicles collected at the same time point from the same animal, controlling for systemic hormonal exposure while allowing direct comparison of follicle-intrinsic differences [5]. Under this design, *CARTPT* mRNA abundance was consistently higher in subordinate follicles than in the co-dominant follicle from the same wave, and this difference was already apparent before subordinate follicles showed any overt morphological evidence of atresia such as pyknotic nuclei or granulosa cell detachment.

This last point is the one that gave the finding its mechanistic weight rather than leaving it as a descriptive curiosity. A transcript that is simply elevated after atresia has visibly begun would be a marker of cell death already underway, of limited interest beyond diagnostics. A transcript elevated before any morphological sign of degeneration, in a follicle that is biochemically declining but still structurally intact, is a much stronger candidate for something contributing causally to that decline, or at minimum tracking a decision point in follicle fate that occurs earlier than previously appreciated [26].

### 4.2. Cellular Localization: Granulosa, Thecal, and Oocyte Compartments

The compartmental distribution of *CARTPT* within the follicle is not a trivial anatomical detail: it constrains, quite directly, what mechanism is biologically plausible. Steroidogenesis in the antral follicle follows the two-cell, two-gonadotropin model: thecal cells, driven by LH acting through the cAMP-protein kinase A pathway, convert cholesterol to androstenedione via the steroidogenic acute regulatory protein and the cytochrome P450 side-chain cleavage and 17α-hydroxylase/17,20-lyase enzymes [27]. This androgen substrate then diffuses across the basement membrane into the avascular granulosa cell compartment, where FSH-driven cAMP-PKA signaling induces CYP19A1, the aromatase enzyme, converting androstenedione and testosterone into estrone and estradiol [28]. Any local regulator proposed to interfere with estradiol output has to act at one of a limited number of points in this sequence: thecal androgen supply, granulosa FSH receptor engagement, the cAMP-PKA cascade itself, or CYP19A1 transcription and enzymatic activity. The compartment in which that regulator is expressed narrows down which of these points is mechanistically accessible to it.

Kobayashi and colleagues localized *CARTPT* expression predominantly to the granulosa cell layer, with detectable but consistently weaker immunoreactivity and mRNA signal in the theca interna, using a combination of in situ hybridization for transcript localization and immunohistochemistry for peptide detection across serial sections of antral follicles at defined stages of development [6]. This granulosa-predominant pattern is mechanistically significant for two reasons. First, it places *CARTPT* in the same cellular compartment as the FSH receptor and the cAMP-PKA-CYP19A1 cascade that FSH drives, rather than in the thecal compartment where it would be positioned to act only indirectly, by limiting androgen substrate supply [29]. A granulosa-localized peptide is positioned for direct, cell-autonomous or paracrine interference with FSH signal transduction itself, which is the mechanism subsequently tested and confirmed at the level of cAMP, ERK1/2, and Akt activation, detailed in Chapter 5. Second, granulosa cells lack their own blood supply until late in folliculogenesis, relying on diffusion across the basement membrane for hormone and nutrient exchange with the thecal vasculature. A peptide produced within this avascular compartment is well positioned to accumulate locally within the follicular fluid microenvironment rather than being rapidly cleared systemically, consistent with its later detection and quantification directly in follicular fluid rather than only in tissue extracts or peripheral circulation [30].

The thecal signal, though weaker, is not negligible, and its interpretation requires some caution rather than dismissal. Austin and colleagues, building on the initial localization data, examined *CARTPT* alongside inhibins and low-molecular-weight insulin-like growth factor binding proteins as a panel of candidate local suppressors of granulosa cell estradiol production, on the premise that estradiol-producing capacity is lost from a subordinate follicle before any visible sign of morphological atresia appears, and that whatever drives this early functional decline must therefore act through paracrine or autocrine signaling within the follicle rather than through systemic gonadotropin withdrawal alone [31]. Granulosa cells harvested from follicles at successive stages of a defined follicular wave spanning the period before, at, and after the point at which growth trajectories of the future dominant and subordinate follicles diverge showed a pattern in which *CARTPT* transcript abundance rose in step with the decline in estradiol-producing capacity, tracking the functional trajectory of the follicle more closely than follicle diameter alone would predict [32]. This design is what allowed the group to argue that *CARTPT* expression precedes, rather than merely accompanies, the loss of steroidogenic function, a temporal relationship that is a necessary, though not sufficient, condition for a causal role.

Reports of CARTPT expression within the oocyte or its surrounding cumulus cell investment are considerably more limited and inconsistent than the granulosa and thecal findings, appearing as a secondary observation in some species-specific studies of ovarian CART biology rather than as a systematically investigated compartment in its own right [33]. Where such signal has been noted, it has not been coupled to any functional readout: no study to date has tested whether oocyte-associated CART peptide influences meiotic competence, cumulus expansion, or oocyte-to-granulosa cell communication via the gap junctions and transzonal projections that normally coordinate these compartments [34]. This is a genuine gap in the literature rather than a settled negative finding, and it leaves open the possibility, currently untested, that *CARTPT* could act not only on the somatic follicular cells but also more directly on the germ cell itself or on the bidirectional oocyte-granulosa signaling axis that governs oocyte developmental competence.

### 4.3. Cross-Species Comparisons and the Absence of CART in the Mouse Ovary

Whether the bovine findings reflect a genuinely conserved feature of follicular biology across mammals and birds, or a peculiarity of the specific model system in which they were first generated, depends on evidence from species beyond cattle, and that evidence, while broadly corroborative, is thinner and less mechanistically developed than the bovine work itself [11].

*CARTPT* expression has been described in porcine ovarian follicles, but the functional picture there is less clear-cut than in cattle. Li and colleagues measured CART peptide effects on FSH-stimulated estradiol production in porcine granulosa cells and found only a non-significant inhibitory trend, leading them to propose that CART is probably not a major local regulator of follicular development in the pig, in contrast to its apparent role in monotocous species such as cattle and sheep [12]. This is a meaningful divergence rather than a minor caveat: it suggests CART’s inhibitory action on FSH signaling may be most pronounced in species that typically select a single dominant follicle, and weaker or absent in polytocous species that ovulate multiple follicles per cycle, a distinction worth testing directly rather than assuming a uniform mechanism across species. In the domestic hen, *CARTPT* transcript and CART immunoreactivity localize predominantly to the thecal layer, with weaker, more diffusely distributed staining in the granulosa layer and occasional signal in the oocyte, a distribution that is not identical to the bovine granulosa-predominant pattern, and CART mRNA abundance differs significantly by follicle size class [34]. Avian folliculogenesis differs from the mammalian pattern in several structural respects, including a markedly stratified thecal layer with distinct internal and external zones and a single, sequentially recruited hierarchy of large yolky follicles rather than the wave-based cohort selection seen in cattle, so some divergence in CART’s cellular distribution between chicken and cow is not, on its own, surprising [2].

The most recent addition to this comparative picture comes from the water buffalo, where Yang and colleagues examined *CARTPT* expression alongside its proposed downstream signaling intermediates directly in atretic versus healthy antral follicles [35]. Atretic follicles showed elevated *CARTPT* and *BAX* transcript levels, with *BAX* encoding a pro-apoptotic member of the BCL-2 family and serving here as a molecular marker of granulosa cell apoptosis, alongside reduced expression of *AKT1*, *CTNNB1* (encoding β-catenin), and *CYP19A1* relative to healthy follicles from the same ovarian pool [35]. When granulosa cells were treated with CART peptide in the presence of FSH in vitro, cell viability and estradiol output fell while apoptotic markers rose, and the authors traced this specifically to suppressed AKT phosphorylation, reduced β-catenin protein stability, and downstream loss of *CYP19A1* transcription, implicating the AKT/GSK3β/β-catenin signaling axis as a mechanistic route distinct from, though potentially convergent with, the cAMP-centered mechanism described in cattle. This is a meaningful extension of the model: it suggests CART’s inhibitory action on granulosa cell steroidogenesis may not run through a single invariant pathway across species, but through at least two identifiable intracellular routes that converge on the same functional endpoint of reduced aromatase expression [36].

Set against this pattern of cross-species presence is a complicating negative observation. *CARTPT* has not, to our knowledge, been reported as constitutively expressed in the mouse ovary under normal physiological conditions, an absence noted in passing within the Michigan State group’s own review of the field, though this point warrants direct confirmation from primary data rather than being taken as definitively established here [11]. If accurate, this would be a substantive complication for the field rather than a minor footnote, for two related reasons. First, it would mean the extensive bovine mechanistic literature spanning granulosa cell binding studies, cAMP and kinase signaling, and follicular wave dynamics cannot be directly extended to the mouse, the species in which the overwhelming majority of reproductive genetics and knockout technology has been developed, leaving a persistent gap between mechanistic depth and genetic tractability. Second, it would mean that the later report of *CARTPT* expression in the mouse ovary under conditions of diet-induced obesity and hyperleptinemia (discussed in Chapter 7) reflects an induced rather than constitutive expression pattern, a distinction worth holding onto rather than treating the mouse and bovine ovarian CART systems as directly equivalent baseline biology that merely differs in degree.

## 5. Molecular Mechanism: CART as an Inhibitory Regulator of FSH Signaling

FSH acts on the granulosa cell through a canonical G protein-coupled receptor mechanism. Ligand binding to FSHR activates Gαs, which stimulates adenylate cyclase and raises intracellular cAMP; cAMP then activates protein kinase A, which phosphorylates CREB and other transcription factors that drive expression of *CYP19A1*, the aromatase gene, along with a broader program of genes involved in granulosa cell differentiation [37]. This is the pathway that converts a gonadotropin signal into an enzymatic capacity to make estradiol, and it is the pathway that the bovine studies place CART directly in the way of.

### 5.1. Effects on cAMP Production and Aromatase Expression

The first direct demonstration that CART interferes with this cascade, rather than merely correlating with follicles that have already stopped responding to it, came from Sen and colleagues, who treated cultured bovine granulosa cells with FSH in the presence or absence of the mature CART peptide [7]. FSH alone produced the expected rise in intracellular cAMP; co-treatment with CART blunted this rise in a manner consistent with interference upstream of, or at the level of, adenylate cyclase activation, rather than at a point further downstream in the cascade. This distinction matters mechanistically [7]. A peptide that reduced cAMP accumulation without affecting FSH receptor binding itself would point to CART acting through a separate, convergent G protein pathway, plausibly the same inhibitory Gi/Go coupling described for CART in neuronal tissue in Chapter 3, rather than through direct competition for the FSH receptor. The downstream consequences followed the pattern predicted by this upstream interference: *CYP19A1* mRNA induction by FSH was attenuated, and estradiol output from the treated cells fell correspondingly [7].

Lv and colleagues extended this in vitro finding into an in vivo physiological context, working within the same bovine follicular wave model described in Chapter 4 [8]. Granulosa cells and follicular fluid collected from subordinate follicles, where *CARTPT* expression is elevated, showed reduced estradiol-producing capacity relative to the co-dominant follicle from the same wave, and this reduction was reproducible by treating granulosa cells from healthy, estradiol-competent follicles with exogenous CART peptide in vitro; the treated cells came to resemble subordinate-follicle granulosa cells in their reduced steroidogenic output [8]. This is a useful piece of internal validation for the model as a whole: it shows that the association between *CARTPT* expression and reduced estradiol capacity observed across natural follicle populations can be reproduced experimentally by manipulating CART alone, holding other follicular variables constant, which strengthens the case that CART is acting as a cause of the estradiol deficit rather than merely a co-regulated marker of it [38].

A further layer of complexity, and one that gives the pathway more explanatory reach than a simple one-directional inhibition, comes from work examining what regulates *CARTPT* expression itself. Gupta and colleagues, investigating WNT signaling as a potentiator of FSH action during dominant follicle selection, found that FSH treatment of bovine granulosa cells increased *CYP19A1*, *CTNNB1*, and several other WNT pathway component transcripts as expected, but had the opposite effect on *CARTPT*: FSH treatment reduced *CARTPT* mRNA, and this suppression was partially reversed by pharmacological inhibition of WNT signaling [39]. Taken together with the inhibitory action of CART on FSH signaling described above, this describes a reciprocal, mutually suppressive relationship rather than a simple upstream-to-downstream inhibition: FSH acts to keep *CARTPT* expression low in the follicle it is supporting toward dominance, partly through WNT-dependent signaling, while CART, where it escapes this suppression and accumulates, acts back on FSH signal transduction to blunt the follicle’s estradiol output. A follicle’s trajectory toward dominance or atresia, under this model, depends on which side of this mutual suppression gains the upper hand early in the wave, which is a more dynamic and arguably more biologically realistic picture than a static association between CART level and follicle fate [39].

### 5.2. Modulation of ERK1/2 and Akt Signaling Kinetics

FSH signaling in the granulosa cell is not confined to the cAMP-PKA axis. FSH also activates the ERK1/2 and PI3K-Akt kinase cascades, both of which contribute to granulosa cell proliferation, survival, and differentiation alongside the steroidogenic program driven by cAMP. Sen and colleagues examined how CART affects these kinase pathways specifically, and the picture that emerged was more subtle than a straightforward blockade [9]. FSH stimulation produced the expected rapid phosphorylation and activation of both ERK1/2 and Akt in bovine granulosa cells; CART co-treatment did not prevent this initial activation from occurring; instead, it shortened its duration. Phosphorylated ERK1/2 and Akt declined more quickly in CART-treated cells than in cells receiving FSH alone, indicating that CART accelerates the termination of kinase signaling rather than blocking its initiation [9].

The mechanism behind this acceleration was traced to mitogen-activated protein kinase phosphatases, a family of enzymes that dephosphorylate and thereby inactivate ERK1/2 and related kinases, acting as the cell’s built-in negative feedback on its own kinase signaling. CART treatment altered both the expression and the degradation kinetics of specific phosphatases within this family in bovine granulosa cells, in a direction consistent with faster inactivation of the FSH-stimulated kinase signal [9]. This is a mechanistically distinct action from the cAMP effect described in Section 5.1, rather than a redundant description of the same event: suppressing cAMP production limits the steroidogenic output of the cell, while accelerating ERK1/2 and Akt inactivation limits the duration of a proliferative and pro-survival signal, and there is no obligatory reason these two actions need to run through the same proximal step in CART’s own signaling. Whether they in fact share a common origin at the level of CART’s Gi/Go-coupled receptor engagement, or represent two separable downstream branches, has not been directly tested.

This kinetic rather than binary mode of action has a practical implication worth stating explicitly: a granulosa cell exposed to CART is not simply unresponsive to FSH. It responds, but for a shorter window, with a proliferative and survival signal that terminates earlier than it otherwise would [15]. Over the several days a follicular wave requires to select a dominant follicle, this kind of quantitative shortening of a signaling window, repeated across many cycles of gonadotropin exposure, is plausibly sufficient to produce a meaningfully different growth trajectory between a follicle with low CART exposure and one with high CART exposure, without requiring FSH signaling to be abolished in the losing follicle at any single time point. This offers a more gradual, cumulative account of follicle divergence than a model in which subordinate follicles simply stop responding to FSH altogether.

**Figure 1:**
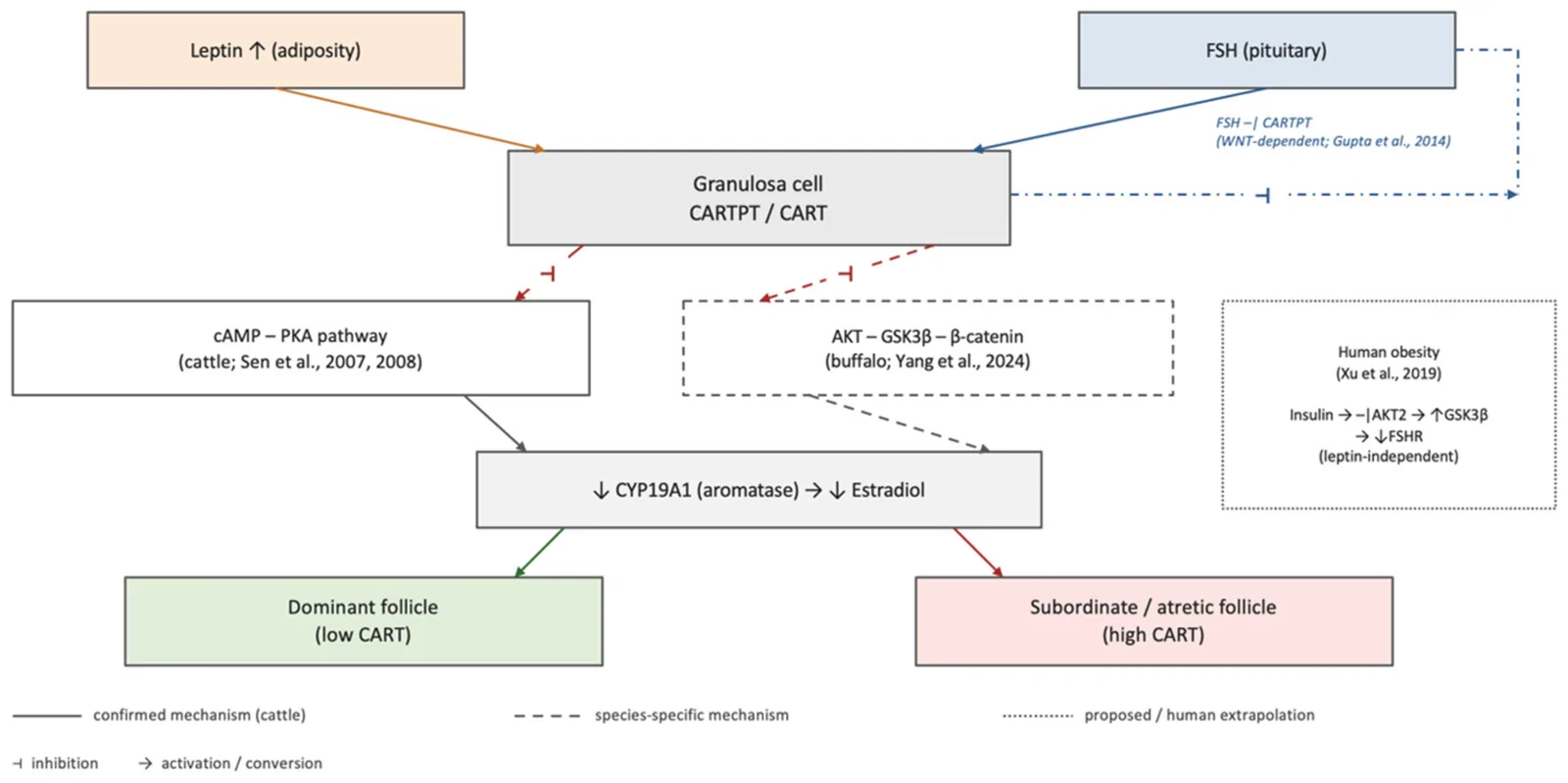
Proposed CARTPT-FSH signaling axis in the ovarian follicle. Leptin induces granulosa cell CARTPT/CART expression, which suppresses FSH-stimulated cAMP-PKA signaling and, in buffalo, the AKT/GSK3β/β-catenin axis, converging on reduced *CYP19A1* (aromatase) expression and estradiol output. FSH reciprocally suppresses *CARTPT* through WNT-dependent signaling, creating a mutual-suppression loop that contributes to divergence between dominant and subordinate/atretic follicles. A parallel, leptin-independent mechanism linking insulin to reduced FSHR expression via AKT2/GSK3β has been reported in obese women undergoing IVF. Solid lines indicate mechanisms confirmed in cattle; dashed lines indicate species-specific findings; dotted lines indicate proposed or human-extrapolated relationships not yet directly tested [9,35,39,40].

A final point connects this chapter back to the receptor pharmacology discussed in Chapter 3. Folger and colleagues, examining CART binding sites on bovine granulosa cells directly, found that binding site density itself changes across the stages of the follicular wave and with hormonal treatment, and that a pharmacological CART signaling inhibitor could partially reverse CART’s suppressive effect on granulosa cell estradiol production [10]. This confirms that the cAMP and kinase effects described above depend on a genuine receptor-mediated interaction rather than a nonspecific action of the peptide, even though, as established in Chapter 3, the molecular identity of that receptor in granulosa cells remains as unresolved as it does in neuronal tissue. Figure 1 summarizes this signaling architecture, distinguishing the confirmed bovine mechanism from the species-specific and human-extrapolated branches discussed in the chapters that follow.

## 6. *CARTPT*, Dominant Follicle Selection, and Atresia

### 6.1. Expression Patterns Across the Follicular Wave

The bovine follicular wave offers an unusually clean natural experiment for studying follicle divergence, because it allows direct, within-animal comparison of follicles exposed to the same systemic hormonal milieu but following different developmental trajectories. A wave begins with the synchronized emergence of a cohort of small antral follicles, roughly similar in size and, presumably, in developmental potential. Within a few days, one follicle, the one that will become dominant, begins to grow measurably faster than its cohort-mates, acquires increasing LH dependence, and eventually reaches the preovulatory stage. The remaining follicles, termed subordinate, plateau and then regress, most showing clear signs of atresia within a week of the point at which growth rates diverge.

*CARTPT* expression tracks this divergence closely, and does so earlier than most other markers examined in the same model. Kobayashi and colleagues in 2004 established the basic pattern: at the point of deviation, when dominant and subordinate follicles are still similar in diameter and show no visible morphological difference, *CARTPT* mRNA is already measurably higher in the subordinate follicles [5]. Kobayashi and colleagues in 2006 extended this observation across a finer-grained time course spanning the period before, at, and after deviation, and found that the rise in *CARTPT* expression in subordinate follicles parallels the fall in their estradiol-producing capacity closely enough in time that the two appear to be part of the same unfolding process rather than sequential, independent events [6]. Lv and colleagues confirmed this pattern using a larger sample of animals and follicular waves, and additionally showed that granulosa cells isolated from subordinate follicles retained a form of “molecular memory” of their status: even after a period in culture, these cells produced less estradiol in response to FSH than dominant-follicle granulosa cells did, a difference partially reproduced by treating dominant-follicle cells with exogenous CART [8].

The timing established across these studies is what gives *CARTPT* its particular interest as a candidate mechanism rather than simply another follicle-status marker. Follicle diameter, by contrast, does not reliably distinguish the future dominant follicle from its largest subordinate competitor until after the deviation point is already underway; several groups working with ultrasound-based staging of follicular waves have noted that growth rate, rather than absolute size at any single time point, is often the more reliable early predictor of which follicle will become dominant [41]. A molecular signal that differs between follicles of nearly identical size, before growth rates have visibly diverged, is mechanistically more interesting than one that simply confirms a difference already obvious from follicle diameter: it raises the possibility that *CARTPT* expression is not merely downstream of the decision about which follicle will dominate, but plays some role in how that decision gets made in the first place.

### 6.2. CARTPT as a Marker Versus a Driver of Atresia

Distinguishing a molecular marker of follicle fate from an active contributor to that fate is not a trivial distinction, and the bovine *CARTPT* literature offers a reasonable, though incomplete, case for the latter interpretation. Four lines of evidence bear on this question, and it is worth being explicit about what each one does and does not establish.

The first is temporal: as described in Section 6.1, elevated *CARTPT* expression in subordinate follicles precedes both the loss of estradiol-producing capacity and any visible morphological sign of atresia. Temporal precedence is a necessary condition for a causal role, but it is not sufficient on its own: many genuinely downstream markers of cell decline also rise before morphological atresia becomes visible under the microscope, simply because biochemical dysfunction generally precedes structural collapse.

The second is interventional, and carries more weight: treating healthy, estradiol-competent granulosa cells with exogenous CART peptide reproduces key features of the subordinate-follicle phenotype, including reduced FSH-stimulated cAMP, reduced *CYP19A1* expression, and reduced estradiol output [7,8]. This kind of gain-of-function experiment is a stronger form of evidence than correlation alone, because it demonstrates that CART is sufficient to produce at least part of the phenotype associated with follicles destined for atresia, under controlled conditions where other variables are held constant. It does not, on its own, establish that endogenous CART is necessary for atresia to occur in vivo; that would require a loss-of-function approach, such as CART neutralization or knockdown within a developing follicular wave, which has not been reported in cattle to date.

The third line of evidence, more recent and drawn from a different species, extends the interventional case to a distinct intracellular pathway. In water buffalo, CART treatment of granulosa cells in the presence of FSH reduced cell viability and increased markers of apoptosis, an effect traced to suppressed AKT phosphorylation, reduced β-catenin stability, and consequent loss of *CYP19A1* transcription [35]. This finding does two things for the overall argument. It adds direct evidence that CART can promote markers of cell death rather than only suppressing steroidogenesis, which is a step closer to “driving atresia” than the bovine estradiol-suppression data alone provide. And by implicating the AKT/GSK3β/β-catenin axis specifically, alongside the cAMP-centered mechanism established in cattle, it suggests CART’s connection to follicle fate may not run through a single invariant intracellular pathway, but through at least two distinguishable routes that converge on the same functional outcome of reduced granulosa cell viability and steroidogenic capacity.

The fourth line of evidence is mechanistic and comes from two companion studies in bovine granulosa cells that each trace CART’s action to a specific downstream effector rather than treating the pathway as a black box. Cheng and colleagues showed that CART downregulates leucine-rich repeat containing 51 (LRRC51) through increased promoter methylation, and that LRRC51 knockdown alone reproduces the CART phenotype: reduced cell viability, reduced estradiol output, lowered StAR, CYP19A1, and cyclin D2 protein, and increased Bim and cleaved caspase-3, while LRRC51 overexpression reversed these changes [36]. In a separate proteomic screen of the same cell system, the same group identified clusterin (CLU) as the most strongly downregulated protein after CART treatment; CLU knockdown alone reduced granulosa cell viability by roughly 19–20%, lowered the anti-apoptotic protein Bcl-XL, raised BAX and cleaved caspase-3, and reduced the cell-cycle proteins cyclin D2, CDK1, and PCNA. Combining CLU knockdown with CART treatment produced no additional effect beyond CLU knockdown alone, a non-additive result the authors interpret as evidence that CLU functions downstream of, rather than merely alongside, CART’s pro-apoptotic action [42]. Together, these two studies begin to convert “CART inhibits granulosa cell function” from a phenomenological description into a pathway with at least two identified molecular effectors, though it remains unclear whether LRRC51 and CLU act in the same cascade, in parallel, or as independent CART-responsive programs, and neither study addresses whether blocking either effector protects follicles from CART’s action within an intact follicular wave, so the loss-of-function gap identified elsewhere in this review for endogenous CART itself remains open.

## 7. Leptin, Obesity, and CARTPT: The Metabolic Axis

### 7.1. The Leptin-CART Parallel Between Hypothalamus and Ovary

Chapter 3 established that CART’s original physiological identity is as a leptin-induced satiety peptide in the arcuate nucleus, where leptin transcriptionally drives CART expression as part of the circuitry that translates adipose tissue mass into a central behavioral signal [4]. The question this chapter takes up is whether an analogous relationship exists locally within the ovary, and if so, whether it offers a plausible route by which systemic metabolic status could influence follicular outcome independent of leptin’s better-documented central effects on gonadotropin-releasing hormone pulsatility and pituitary function.

The molecular logic behind proposing such a parallel rests on the leptin receptor’s signaling behavior. Leptin acts through the long-form leptin receptor, Ob-Rb, a class I cytokine receptor that signals through constitutively associated Janus kinase 2 (JAK2); ligand binding induces receptor dimerization and JAK2 trans-autophosphorylation, which creates docking sites for STAT3, and phosphorylated STAT3 dimerizes and translocates to the nucleus to drive target gene transcription [43]. That this cascade specifically drives CART induction, rather than simply co-occurring with it, has direct experimental support: intracerebroventricular leptin administration in rats increases arcuate-median eminence mRNA for STAT3, for CART, and for suppressor of cytokine signaling 3 (SOCS3, the JAK2-STAT3 pathway’s own negative feedback regulator) together and in parallel, with CART mRNA rising by roughly 100% relative to control [44]. A separate study using systemic leptin administration found that CART-immunoreactive neurons in the arcuate nucleus frequently co-localize with phosphorylated STAT3, reinforcing at the anatomical level that CART-expressing neurons are among those directly engaged by leptin-JAK2-STAT3 signaling rather than responding indirectly through an intermediary cell population [45]. Whether granulosa cells possess a comparable Ob-Rb-JAK2-STAT3 cascade capable of driving *CARTPT* transcription has not been directly tested; Ob-Rb is expressed on granulosa cells and leptin-activated STAT3 signaling has been documented there in other contexts, which makes the parallel plausible, but the induction of *CARTPT* specifically through this pathway in the ovary remains an extrapolation from the hypothalamic mechanism rather than a demonstrated finding.

This remains, however, an inference built from a plausible shared mechanism rather than a demonstrated one. Several gaps separate the hypothalamic and ovarian contexts that are easy to elide if the parallel is stated too quickly. Leptin signaling in the hypothalamus is subject to extensive negative feedback, most notably through suppressor of cytokine signaling 3 (SOCS3), which is itself induced by STAT3 activation and limits further JAK2-STAT3 signaling, a feedback loop whose existence and strength in granulosa cells has not been characterized in the context of CART induction specifically [46]. Leptin resistance, a hallmark of chronic obesity in the hypothalamus and a major reason why simply elevating circulating leptin does not straightforwardly suppress appetite in obese individuals, is a phenomenon defined and studied almost entirely in neurons [47]. Granulosa cells develop an analogous blunted responsiveness to chronic hyperleptinemia, and how that would affect ovarian CART induction over the course of sustained obesity rather than an acute experimental exposure, is an open question with direct relevance to the human data discussed in Chapter 8. Treating the hypothalamic and ovarian leptin-CART relationships as interchangeable, rather than as structurally similar but independently regulated systems, risks overstating how well-established the ovarian side of this parallel actually is.

### 7.2. Evidence from Diet-Induced Obesity Models

Ma and colleagues tested this hypothesis directly, using diet-induced obesity in mice as a model of chronic hyperleptinemia [12]. The experimental design proceeded in stages, each addressing a distinct link in the proposed causal chain rather than establishing the whole pathway at once.

The first stage was observational. Mice maintained on a high-fat diet developed the expected obese phenotype, with elevated body weight, adiposity, and circulating leptin relative to mice on standard chow. Granulosa cells isolated from the ovaries of these obese mice showed increased *CARTPT* transcript abundance relative to lean controls, establishing an association between diet-induced obesity and local ovarian CART expression [12]. This finding carries a particular significance beyond the immediate obesity question, given the discussion in Chapter 4: *CARTPT* had not previously been reported as expressed in the mouse ovary under standard physiological conditions, which raised a genuine question about whether the mouse was simply a poor model for studying ovarian CART biology at all. Its appearance here, specifically and apparently only under a diet-induced obesogenic state, reframes that earlier negative finding: the mouse ovary may not lack the capacity for *CARTPT* expression altogether, but rather requires a sufficiently strong inductive signal, such as chronic hyperleptinemia, to activate a pathway that remains largely silent under normal metabolic conditions. This is a meaningfully different claim from either “mice express *CARTPT* like cattle do” or “mice cannot express *CARTPT* at all,” and it is worth holding onto that distinction rather than collapsing it into either extreme [12].

The second stage moved from association toward causation. Ma and colleagues administered exogenous leptin to lean, normal-weight mice and found that this alone was sufficient to raise granulosa cell *CARTPT* expression, without requiring the broader metabolic and dietary changes that accompany diet-induced obesity [12]. This is an important control: it isolates leptin itself as sufficient to drive the CART induction, rather than some other correlate of high-fat feeding such as altered lipid availability, changes in insulin sensitivity, or shifts in gut microbiota (all of which also change with diet-induced obesity). It does not, on its own, establish that leptin is necessary for the effect seen in diet-induced obesity, which would require a leptin-neutralization or receptor-knockout approach within the obese model itself; the experiment demonstrates sufficiency of leptin administration in lean animals, a related but distinct question from necessity within the obese phenotype.

The third stage connected this induced CART expression to functional consequences already established in the bovine literature. Both the diet-induced obese mice and mice treated directly with exogenous CART peptide, independent of any dietary manipulation, showed a convergent phenotype: reduced granulosa cell cAMP accumulation in response to gonadotropin stimulation, reduced aromatase expression, and impaired ovulation relative to lean or untreated controls [12]. This convergence is the central argument of the paper. Two independent routes to elevated ovarian CART exposure, one through diet and endogenous leptin and one through direct peptide administration, arrived at a similar downstream reproductive phenotype, which is a stronger form of evidence than either observation alone would provide [12]. It suggests that at least part of the reproductive impairment associated with diet-induced obesity in this model runs through the same local CART-FSH interference mechanism characterized biochemically in the bovine granulosa cell studies of Chapter 5, rather than operating exclusively through leptin’s well-established central effects on gonadotropin-releasing hormone pulsatility and pituitary gonadotropin output.

Several qualifications deserve to be stated explicitly rather than left implicit, since the strength of this evidence is easy to overstate in summary form. This is a single published study, in a single species, under one specific dietary intervention protocol. It demonstrates that the leptin-CART-FSH pathway can operate in the mouse ovary under experimentally induced obesity. It does not demonstrate that this pathway is the dominant, or even a major, contributor to obesity-related subfertility. Other mechanisms operate in parallel and remain independently documented: leptin’s central actions on the hypothalamic-pituitary axis, insulin resistance and hyperinsulinemia, chronic low-grade inflammation, and altered adipokine profiles beyond leptin itself [48,49,50]. Our study does not attempt to partition the relative contribution of each. The induced, rather than constitutive, nature of *CARTPT* expression in this model also means that extrapolating from lean-mouse or standard-diet physiology to obese-patient physiology requires treating obesity itself as the relevant experimental condition, not as an incidental variation on a baseline that already includes ovarian CART activity, a distinction that matters directly when this mouse model is used, as it is in Chapter 8, to motivate the interpretation of a single human correlational study. Table 1 summarizes the cross-species evidence for *CARTPT*-mediated regulation of FSH signaling and follicular fate discussed in Chapters 4 through 7, arranged to make the divergence between monotocous and polytocous species, and between preclinical and human evidence, immediately visible.

### 7.3. Human Evidence from Bariatric Intervention

The mouse diet-induced obesity model has a human analogue in bariatric surgery, where adiposity and circulating leptin fall sharply within months. Voros and colleagues followed 29 morbidly obese, infertile women (BMI ≥ 40 kg/m^2^) before and six months after sleeve gastrectomy, measuring granulosa/follicular-fluid CART and leptin transcript alongside FSHR Ser680Asn genotype and reproductive hormones [52]. Mean BMI fell by 16.03 kg/m^2^ over the six months, and FSH levels differed significantly by FSHR genotype both before and after surgery, but the change in CART and leptin expression over the same period was independent of FSHR genotype (*p* = 0.4275 and *p* = 0.6387, respectively). The study did not report whether CART or leptin expression rose or fell in absolute terms following surgery, so this should be read as evidence that genotype does not gate the metabolic response of ovarian CART to weight loss, not as a direct replication of the BMI correlation report in Section 8.1.

## 8. Human Data: CARTPT, Follicular Fluid Leptin, and IVF Outcomes

### 8.1. Direct Evidence: CARTPT and BMI in Women Undergoing IVF

The mouse experiments described in Chapter 7 motivated Ma and colleagues to look for the same relationship directly in human tissue [12]. Granulosa cells and follicular fluid were collected from women undergoing oocyte retrieval as part of standard IVF treatment. *CARTPT* transcript abundance in granulosa cells was quantified by quantitative RT-PCR, and CART peptide concentration in follicular fluid was measured by immunoassay, alongside each patient’s body mass index. Both measures correlated positively with BMI, extending the mouse finding of leptin-induced granulosa cell *CARTPT* expression to a human cohort under standard clinical IVF conditions rather than an experimental diet-induced obesity model [12].

What makes this more than a simple replication of the mouse finding is the molecular continuity it implies across the whole pathway traced in this review. The bovine mechanistic work in Chapter 5 established that CART acts in granulosa cells by suppressing FSH-stimulated adenylate cyclase activity, reducing cAMP accumulation, and consequently lowering PKA-dependent phosphorylation of CREB and other transcription factors that drive *CYP19A1* induction. If human granulosa cells respond to CART through the same Gi/Go-coupled mechanism inferred from both the neuronal pharmacology in Chapter 3 and the bovine granulosa cell data in Chapter 5, then a positive correlation between BMI and follicular fluid CART peptide is not merely a descriptive biomarker association: it implies that a larger proportion of granulosa cells in higher-BMI patients are, at the moment of retrieval, under active suppression of their cAMP-PKA-aromatase axis, with corresponding downstream consequences for local estradiol output that this single study did not itself measure. This is an inference built on the assumption that the human ovarian CART receptor pharmacology mirrors the bovine and neuronal data, an assumption that, as established in Chapter 3, cannot currently be confirmed at the receptor level in any species, human included.

The scope of the Ma et al. finding needs to be stated carefully for this same reason [12]. The study establishes an association between adiposity and ovarian *CARTPT* expression in women. It does not extend to measuring any of the specific molecular intermediates, such as cAMP, phosphorylated CREB, *CYP19A1* transcript, aromatase protein, or granulosa cell estradiol output, that would confirm the pathway is functionally engaged in these human samples rather than simply co-expressed alongside it. It also does not link *CARTPT* levels to oocyte yield, fertilization rate, embryo quality, or live birth in the same cohort, so the chain connecting elevated CART to an actual reproductive endpoint in humans remains, at present, an extrapolation from the animal mechanistic data rather than a demonstrated clinical relationship. Sample size and patient characteristics beyond BMI were not detailed to an extent that would allow assessment of how representative the cohort is of the broader IVF population, and independent replication has not yet been reported. The finding is best read as establishing biological plausibility at the level of gene and peptide expression that the pathway characterized mechanistically in cattle and functionally in mice can be detected operating in the human ovary rather than as evidence that *CARTPT* already functions as a validated marker of any clinical outcome, or that its downstream molecular consequences have been confirmed in human tissue.

Voros and colleagues addressed exactly this gap in a cohort roughly twenty-fold larger than Ma et al.’s [38]. In 94 women undergoing IVF across four ovulation induction protocols (HMG, HMG/hCG, rFSH, rFSH/hCG), follicular fluid CART, quantified by RT-PCR, correlated negatively with the number of follicles exceeding 12 mm (r = −0.251, *p* = 0.018), total oocyte count (r = −0.247, *p* = 0.019), and oocyte maturity (r = −0.212, *p* = 0.048). This is, to date, the only human data connecting CART directly to an ovarian response or oocyte-quality endpoint rather than to BMI alone, and it is directionally consistent with the inhibitory role CART plays in the bovine and buffalo mechanistic literature: higher follicular fluid CART tracked with fewer, less mature oocytes. CART did not correlate with reproductive hormones across the full cohort, and showed a positive association with FSH only within the rFSH/hCG subgroup, a protocol-specific finding that remains unexplained mechanistically. Carriers of the FSHR Ser680Ser genotype had higher CART levels than Ser/Asn or Asn/Asn carriers, raising the possibility that FSHR polymorphism status modulates follicular CART biology, though this was not the study’s primary endpoint and requires independent replication.

### 8.2. The Broader and Inconsistent Follicular Fluid Leptin Literature

Because leptin sits upstream of CART in the proposed pathway, and because leptin itself has been measured in human follicular fluid far more extensively than CART has, the leptin-IVF outcome literature offers a useful, if indirect, test of whether the broader metabolic signal this pathway depends on actually predicts reproductive outcomes in women [53]. Before turning to that inconsistency, it is worth noting that leptin has an independently documented, CART-independent route of interference with FSH signaling in the granulosa cell, which matters for interpreting why the human data might be noisy even if the underlying biology is real [54]. Zachow and Magoffin showed, in rat granulosa cells, that leptin impairs the synergistic action of insulin-like growth factor-I on FSH-dependent estradiol-17β production, a mechanism operating through the IGF-1 arm of granulosa cell steroidogenesis rather than through CART or the cAMP-PKA axis directly [54]. This means that in human follicular fluid, leptin is positioned to interfere with granulosa cell estradiol output through at least two molecularly distinct routes: directly, via IGF-1 synergy disruption, and indirectly, via *CARTPT* induction as described in Chapter 7. Any given clinical study measuring only leptin concentration against a reproductive outcome cannot distinguish which of these routes, if either, is operating in a given patient, let alone quantify their relative contribution.

Against this more complicated mechanistic backdrop, individual clinical studies have reported positive, negative, and null associations between follicular fluid leptin and IVF outcomes, often in comparably sized cohorts using similar assay methods. De Placido and colleagues found that follicular fluid leptin concentration was a better predictor of oocyte fertilization than follicle diameter in a cohort of normogonadotrophic women, with a leptin concentration above 20.25 ng/mL associated with a fertilization rate of 85.7%, compared with 16.7% below that threshold [55]. Hong and colleagues, using a cohort stratified by ovarian reserve, found that follicular fluid leptin predicted oocyte maturity with reasonable discriminative accuracy, though the relationship depended on where a given patient fell on the anti-Müllerian hormone spectrum rather than holding uniformly across all reserve categories [56]. Against these positive findings, Welt and colleagues, examining leptin alongside its soluble receptor across a comparable number of individual follicles, found no relationship between follicular fluid leptin and markers of oocyte quality, fertilization, or embryo grade, despite leptin and its receptor being strongly interrelated with each other and with other intrafollicular hormones, a result consistent with the possibility that leptin’s soluble receptor buffers free leptin availability to a degree that varies enough between patients to obscure a dose-dependent relationship with outcome [57].

This pattern of inconsistency is not simply a matter of a few outlying studies; it holds up under formal synthesis. Jafarpour and colleagues conducted a systematic review and meta-analysis pooling standardized mean differences in serum and follicular fluid leptin across fourteen eligible studies comparing women who achieved pregnancy after IVF or ICSI with those who did not, and found no statistically significant difference in leptin concentration, in follicular fluid or serum, at either oocyte retrieval or the time of triggering ovulation [58]. Their conclusion was not that leptin is irrelevant to reproductive outcome, but that its relationship to any single IVF endpoint is not consistent or strong enough across the accumulated literature to function as a standalone predictive marker, and that heterogeneity in patient selection, assay platform, and outcome definition likely obscures whatever true relationship exists beneath study-to-study noise that, given the dual IGF-1 and CART-mediated routes described above, may partly reflect genuine underlying mechanistic heterogeneity between patients rather than purely methodological variation.

### 8.3. FSH Receptor Expression and Insulin Signaling in Obesity-Related Infertility

Leptin and CART are not the only, or even necessarily the dominant, molecular route by which obesity is thought to impair granulosa cell responsiveness to FSH, and a full picture of the human data requires placing the CART pathway alongside this alternative mechanism rather than in isolation [59]. Xu and colleagues examined FSH receptor expression directly in granulosa cells from women who were overweight or obese undergoing IVF, compared with normal-weight controls, and found both *FSHR* mRNA and FSHR protein reduced in the higher-BMI groups [40]. Rather than tracing this to leptin, the study identified insulin and glucose, both positively correlated with BMI in fasting blood and in follicular fluid in this cohort but notably not correlated with follicular fluid leptin level in the same patients, as the operative upstream signal.

The mechanism proposed operates through the same GSK3β–β-catenin node described for CART action in buffalo, but read out at a different phosphorylation site. Treating human granulosa-like tumor cells with insulin reduced estradiol production and reduced phosphorylation of Akt2, while increasing phosphorylation of glycogen synthase kinase 3 (GSK3) at Tyr216, its activating autophosphorylation site, rather than the inhibitory Ser9 residue that Akt targets in the buffalo CART studies. Because Tyr216 phosphorylation reflects GSK3 activity rather than its suppression, this is functionally consistent with, not inverse to, the buffalo pattern: reduced Akt signaling leaves GSK3β less restrained in both cases, and in both cases the downstream consequence is reduced β-catenin [40]. Here, insulin’s effect on this same intracellular axis, mediated through reduced Akt2 activity and increased GSK3 activity, was associated with decreased *FSHR* transcription itself, upstream of the receptor’s ligand-binding and signal-transducing function altogether, a mechanistically prior point of interference compared with CART’s action on post-receptor cAMP and kinase signaling [40]. Pharmacological inhibition of GSK3β in granulosa cells from obese patients partially restored β-catenin activity and *FSHR* expression, along with increased expression of insulin receptor substrate 1 and PI3K, supporting a causal role for this GSK3β-β-catenin node in the observed *FSHR* deficit.

The explicit dissociation from follicular fluid leptin in this study is worth underscoring, because it clarifies rather than complicates the overall picture developed across this review. It indicates that at least two molecularly separable, obesity-associated mechanisms converge on impaired granulosa cell responsiveness to FSH: a leptin-CART axis acting primarily on post-receptor cAMP-PKA and ERK1/2-Akt signaling downstream of an intact FSH receptor, as established in Section 5, Section 6 and Section 7 and an insulin-GSK3β-β-catenin axis acting further upstream on FSHR expression itself, largely independent of leptin or CART [40]. These mechanisms are not mutually exclusive within a single obese patient. A granulosa cell exposed to both elevated leptin/CART and elevated insulin could plausibly experience compounded impairment: reduced receptor density together with blunted signaling through whatever receptors remain, though no study to date has examined both pathways simultaneously in the same patient samples to test for such an additive or synergistic effect, a gap consistent with the broader observation that insulin resistance and hyperleptinemia are typically treated as parallel, independently reviewed contributors to obesity-related reproductive dysfunction rather than as an integrated mechanism [60]. Table 2 turns to the human clinical literature specifically, situating the direct CARTPT findings within the larger and more inconsistent body of work on follicular fluid leptin discussed in Chapter 8.

## 9. Clinical and Research Implications

The evidence traced across this review does not yet support any direct clinical application of *CARTPT* in reproductive medicine, and it is important to state that plainly before discussing what it might eventually support. No CART-based test, threshold, or intervention has been validated in a clinical trial, and a recent cohort has linked follicular fluid CART to oocyte yield and maturity [38], though this has not yet been tested against fertilization, embryo quality, or live birth, nor independently replicated. What the evidence does support is a more circumscribed set of near-term possibilities, each contingent on further data this review identifies as missing, discussed in full in Chapter 11.

The most direct potential application is as a follicular fluid biomarker in women undergoing ovarian stimulation, particularly those with obesity-related infertility where FSH responsiveness is already known to be variable and difficult to predict from BMI or antral follicle count alone. If CART peptide concentration in follicular fluid proves, in future cohorts, to correlate with oocyte or embryo outcomes more consistently than follicular fluid leptin has, it could in principle supplement existing biomarkers used to individualize gonadotropin dosing, given CART’s more specific, receptor-mediated position downstream of leptin, as discussed in Chapter 8. This remains speculative rather than actionable at present: it would require, at minimum, a prospective cohort measuring *CARTPT* alongside conventional outcome measures, which has not yet been conducted.

A second, related possibility follows from the phenotype-heterogeneity discussion in Chapter 11. If obesity-related IVF impairment does in fact stratify into a leptin-CART-predominant subgroup and an insulin-*GSK3β*-*FSHR*-predominant subgroup, as the discrepancy between the two human studies discussed there suggests but does not establish, this would argue for phenotyping obese IVF patients on both leptin and insulin resistance status rather than treating obesity as a single undifferentiated risk category. Such stratification could eventually inform which patients might benefit more from insulin-sensitizing pretreatment versus other interventions, though this again rests on a hypothesis this review has raised rather than a finding it can report. For research rather than immediate clinical purposes, the CART pathway offers a mechanistically specific target for interventional studies that the broader leptin literature, given its inconsistency, cannot easily provide. A pharmacological CART signaling inhibitor, already used experimentally in bovine granulosa cells, represents a concrete tool for testing whether blocking this pathway in vivo rather than only in vitro measurably improves FSH responsiveness or estradiol output in follicles otherwise destined for atresia, which would be a meaningful step toward establishing causality rather than correlation in a physiologically intact system.

## 10. Knowledge Gaps and Future Directions

Several gaps recur across this review and are worth consolidating rather than leaving scattered across individual chapters. The most fundamental is the unresolved ovarian CART receptor: every mechanistic claim in Chapters 5 through 7, in every species examined, still rests on a pharmacological signature rather than a receptor confirmed in that tissue. The field has moved since the exclusion of GPR160: Lindqvist and colleagues identified GPR162 as a genuine CART receptor in pancreatic beta and alpha cells using binding, knockdown, and imaging approaches that go well beyond the earlier candidate-gene work [19], but that identification has not been extended to the ovary. The open question is therefore narrower than a search for any CART receptor at all: whether GPR162 specifically is expressed on granulosa cells and accounts for the CART binding sites already described there. Resolving it would allow the granulosa cell binding sites described to be assigned a molecular identity, and would make possible the kind of receptor-targeted pharmacology that has not yet been applied to the ovarian pathway.

A second gap is the absence of loss-of-function data within an intact follicular wave. Every causal claim about CART driving, rather than merely marking, follicle selection rests on gain-of-function experiments adding exogenous CART to otherwise healthy follicles. Whether neutralizing or knocking down endogenous CART within a developing wave rescues subordinate follicles from atresia has not been tested in any species, and this remains the single most direct experiment that could move the field from strong correlation to demonstrated necessity.

Third, the cross-species picture needs systematic rather than incidental expansion. The monotocous-versus-polytocous hypothesis proposed in Chapter 11 generates a specific, testable prediction that CART’s inhibitory effect should track reproductive strategy rather than phylogenetic distance from cattle, but confirming or refuting it requires functional data from additional species deliberately chosen to span this axis, rather than the current, largely opportunistic sample. Within this, the mouse remains a particular puzzle: whether *CARTPT* is genuinely silent under baseline conditions and inducible only under metabolic stress, as this review has interpreted the evidence, or whether baseline expression has simply not been looked for carefully enough, has not been resolved.

Fourth, the proposed convergence between the cAMP-PKA and AKT/GSK3β mechanisms, and its complication by Axin-mediated pathway insulation, has not been tested directly in granulosa cells of any species. Measuring Axin-GSK3β complex composition alongside cAMP and Akt signaling in the same CART-treated cells would settle whether the two mechanistic threads traced in this review genuinely converge or remain functionally separate. Finally, and most consequentially for eventual clinical relevance, no study has measured *CARTPT*, follicular fluid leptin, and insulin resistance status together in the same women undergoing IVF. This is the most direct route to testing the phenotype-stratification hypothesis raised in Chapter 11, and it would require nothing beyond applying assays already validated individually to a single, adequately characterized cohort.

Table 3 summarizes these five gaps alongside the specific experiments that would resolve each one.

## 11. Discussion

Before turning to how these studies interact with one another, it is worth summarizing which findings this chapter draws together. The core mechanistic claim rests on the bovine work establishing CART’s suppression of cAMP-PKA and ERK1/2-Akt signaling in granulosa cells; the expression studies linking *CARTPT* abundance to follicle status within the follicular wave; the interventional work confirming this relationship by direct CART administration; the characterization of CART binding site regulation across the wave; and the finding that FSH itself reciprocally suppresses *CARTPT* through WNT signaling. This mechanism was extended to buffalo through a distinct AKT/GSK3β/β-catenin route, while the corresponding porcine study found no significant effect. On the human side, one study reported the only direct CARTPT-BMI correlation in women undergoing IVF, a meta-analysis synthesizing fourteen studies on follicular fluid leptin and IVF outcome found no significant association, and a separate study identified a parallel, leptin-independent insulin-GSK3β mechanism suppressing FSHR expression in obesity; a fourth, larger cohort linked follicular fluid CART directly to oocyte yield and maturity in women undergoing ovarian stimulation. The sections that follow bring in additional literature on GSK3β biochemistry, on the mathematics of follicle competition, on reproductive strategy evolution, and on metabolic phenotype heterogeneity to examine what happens when all of these studies are read against each other rather than as an independent list of findings.

The two intracellular mechanisms our research has traced are not obviously the same pathway described twice in different species, but they are not obviously independent either, and the relationship between them deserves more scrutiny than treating them as a simple list of “mechanism one, mechanism two.” Sen et al. (2007, 2008) established, in cattle, that CART suppresses cAMP-PKA signaling downstream of the FSH receptor [7,9]. Yang et al. (2024) established, in buffalo, that CART suppresses AKT phosphorylation and destabilizes β-catenin, acting through the AKT/GSK3β/β-catenin axis [35].

There is a documented biochemical point of intersection between these two findings, rather than merely a speculative one. Fang et al. (2000) showed, in a general cell-biology context using Rat1 and NIH 3T3 cells, that PKA directly phosphorylates GSK3β at serine 9, the same inhibitory residue targeted by Akt, physically associating with and inactivating the kinase through a route they demonstrated to be independent of PI3K activity, since PKA-mediated GSK3β phosphorylation persisted even when PI3K was pharmacologically blocked [61]. If this PKA-GSK3β link operates in granulosa cells, then CART’s suppression of cAMP-PKA signaling, as Sen et al. (2007, 2008) describe it, would be expected to reduce PKA-mediated Ser9 phosphorylation of GSK3β, thereby increasing GSK3β activity, the same net direction as CART’s more direct suppression of Akt-mediated GSK3β inhibition that Yang et al. (2024) report in buffalo [7,9,35]. On this research, the bovine and buffalo mechanisms are not two unrelated findings but two different upstream inputs converging on a shared downstream node, which would make the overall CART mechanism considerably more robust than either study alone suggests: even in a follicle where one input pathway is weaker for some reason, the other could still drive GSK3β activation and the downstream loss of β-catenin and aromatase.

This convergence is complicated, however, by a more recent finding that argues against treating it as straightforward. Gavagan et al. (2023) showed, using biochemical reconstitution and cell culture assays, that the pool of GSK3β engaged in Wnt/β-catenin signaling, the specific pool relevant to *CYP19A1* regulation via β-catenin in the buffalo model, is allosterically protected from Ser9 phosphorylation by the scaffold protein Axin, which sequesters Wnt-associated GSK3β and prevents accumulation of the inactive, Ser9-phosphorylated form within the Axin-GSK3β destruction complex specifically, even while PKA and Akt continue phosphorylating GSK3β elsewhere in the cell [62]. This finding, notably, resolves a puzzle that had stood unexplained since Fang et al.’s original 2000 paper: if PKA and Akt globally inactivate GSK3β, it had been unclear why growth factor and cAMP signaling do not also cross-activate Wnt/β-catenin signaling as a side effect [61]. Gavagan et al.’s answer is that they largely do not, because Axin insulates the relevant pool [62]. If an analogous Axin-scaffolded pool exists in granulosa cells, CART’s suppression of cAMP-PKA signaling in the cattle model might leave the Wnt/β-catenin-associated GSK3β pool relatively untouched, which would argue against, rather than for, a clean mechanistic convergence between the bovine cAMP pathway and the buffalo AKT/β-catenin pathway. Neither Sen et al. (2007) nor Yang et al. (2024) measured Axin scaffolding or examined which GSK3β pool their respective interventions affected, so this remains an open, testable question rather than a resolved one; but it means the convergence hypothesis, however biochemically plausible in outline, cannot be assumed to hold at the level of the specific β-catenin pool that matters for aromatase regulation, and a direct test measuring Axin-GSK3β complex composition in CART-treated granulosa cells from both species would be needed to settle it either way [7,35].

This same AKT/GSK3β/β-catenin node reappears a third time in this review, in a completely different context. Xu et al. (2019), working in human granulosa cells, showed that insulin reduces Akt2 phosphorylation and increases GSK3β phosphorylation at Tyr216, its activating autophosphorylation site, rather than the inhibitory Ser9 residue that Fang et al. (2000) and Gavagan et al. (2023) describe [35,40,62]. The raw phosphorylation signal moves in the opposite direction from those Ser9 studies because a different, functionally opposite site is being measured, not because insulin drives GSK3β toward inactivation: like reduced Ser9 phosphorylation, increased Tyr216 phosphorylation reflects a net gain in GSK3β activity, so the insulin and CART/PKA mechanisms converge on the same functional outcome, more active GSK3β and less β-catenin, even though they are read out through opposite-direction signals at different residues [40,61,62]. This raises a genuine, still-open question about mechanism rather than functional direction: are Ser9 dephosphorylation and Tyr216 phosphorylation co-regulated within the same GSK3β pool by a shared upstream event, or do CART/PKA signaling and insulin/Akt2 signaling act on GSK3β through fully independent regulatory routes that happen to converge on the same endpoint? A shared node would point to Ser9-linked inhibition acting as the common switch, as in Fang et al. (2000) and the Akt arm of Yang et al. (2024) [35,61]. Independent routes would instead mean insulin/Akt2 and CART/PKA signaling reach the same *FSHR* or *CYP19A1* deficit through separate molecular switches, which matters for treatment: a shared node would suggest a single pharmacological point of intervention for both hyperleptinemia-driven CART elevation and hyperinsulinemia, while independent routes would mean the two obesity-associated insults need to be targeted separately rather than expected to resolve together. Gupta et al.’s (2014) finding that FSH itself suppresses *CARTPT* expression through WNT-dependent signaling changes how the earlier expression-pattern studies in this review should be read [39]. Kobayashi et al. (2004, 2006) established that *CARTPT* rises in subordinate follicles before morphological atresia is visible, most naturally read as CART accumulating and then actively suppressing an otherwise FSH-responsive follicle [5,6]. Gupta et al.’s (2014) finding complicates a purely one-directional reading: if FSH keeps *CARTPT* low in the follicle it supports, a follicle receiving a marginally weaker FSH signal for any reason would show reduced WNT-mediated suppression, allowing *CARTPT* to rise, which would then further blunt FSH responsiveness through the mechanisms Sen et al. describe, a mutual-suppression loop rather than a single linear cause [39]. Lv et al.’s (2009) interventional data showing that exogenous CART applied to healthy, FSH-responsive granulosa cells is sufficient to reproduce the subordinate-follicle phenotype is what tips the balance back toward CART as an active cause rather than a passive consequence, since Lv et al.’s experiment removes the follicle’s own prior FSH exposure history from the equation entirely [8]. But Gupta et al.’s (2014) finding means that in vivo, within an intact follicular wave, the causal arrow almost certainly runs in both directions simultaneously [39].

This kind of reciprocal-suppression architecture is precisely the structure that Lange et al. (2019) formalized mathematically, independent of any specific molecular mechanism, in their ordinary differential equation model of bovine follicular competition [63]. Working purely from follicle growth and decline data obtained by ultrasound, without reference to CART or any single named signaling pathway, Lange et al. (2019) showed that a competitive interaction term among follicles in which each follicle’s growth suppresses its neighbors’ growth in proportion to its own size is sufficient to reproduce the selection of exactly one dominant follicle from an initially similar cohort, and that this outcome holds robustly across a range of parameter values without requiring pre-existing differences between follicles at the outset [63]. Their model also demonstrated something specific and useful for interpreting the CART literature: the number of follicles that ultimately become dominant is determined by the ratio of follicular growth rate to competition strength, and is mathematically independent of how many subordinate follicles are present in the starting cohort. Read alongside Gupta et al.’s (2014) molecular finding, Lange et al.’s (2019) model offers a plausible formal description of what the FSH-*CARTPT* feedback loop could look like in aggregate: CART-mediated suppression, reinforced by FSH’s own suppression of *CARTPT* in whichever follicle currently has the upper hand, is one concrete molecular candidate for the competition-strength parameter that Lange et al.’s (2019) model requires but does not itself specify biologically [39,63]. It is worth being precise about the limits of this connection: Lange et al.’s (2019) model was fit to bovine ultrasound growth-rate data, not to any molecular measurement, and was not built with CART in mind [63]. The correspondence proposed here is an interpretive alignment between a systems-level model and a molecular mechanism described in separate studies, not a validated instantiation of one within the other. No study has yet parameterized Lange et al.’s (2019) competition model using CART expression or signaling data directly, which would be a natural and fairly direct way to test whether the molecular mechanism can quantitatively account for the growth dynamics the mathematical model already fits well [63].

Li et al.’s (2018) non-significant finding in the pig is not straightforwardly comparable to the positive findings that Kobayashi et al. (2004), Sen et al. (2007, 2008), and Yang et al. (2024) report in cattle and buffalo, and treating it as a simple failure to replicate understates what is actually a more informative discrepancy [5,7,9,35,51]. The bovine and buffalo studies all operate within a reproductive strategy in which a single follicle is actively selected against a cohort of size-matched competitors within a defined follicular wave, the biological context in which a follicle-suppressing signal, and the kind of competitive dynamics Lange et al. (2019) modeled above, would be expected to matter most, because the fate of subordinate follicles is a binary win-or-lose outcome relative to the dominant follicle [63]. Pigs, as a highly polyovulatory species, do not select a single dominant follicle in this way; multiple follicles are typically supported to ovulation simultaneously.

This distinction between reproductive strategies is not merely a descriptive convenience borrowed for this discussion; it reflects a documented evolutionary divergence at the genetic level. Ahn et al. (2014), in a comparative genomic analysis spanning eight monotocous and seven polytocous mammalian species, identified a set of genes showing evidence of differential, lineage-specific adaptive evolution between the two reproductive strategies, several of which are directly or indirectly related to ovulation, most notably an expansion of the EPH receptor gene family specifically in monotocous lineages, a family independently implicated in litter-size regulation in pig genetics research [64]. While *CARTPT* itself was not among the genes Ahn et al. identified as differentially evolved in their specific comparative screen, their broader finding establishes, at the level of the genome rather than any single mechanism, that monotocous and polytocous mammals have undergone measurably different evolutionary pressure on genes governing ovulation number. This provides independent, genome-level support for treating the porcine CART result as reflecting a genuine difference in reproductive strategy-relevant biology, rather than as an isolated experimental anomaly restricted to CART signaling.

Li et al.’s (2018) own interpretation that CART is probably not a major local regulator in the pig is consistent with this broader evolutionary framing: the mechanism CART appears to serve in cattle and buffalo, enforcing single-follicle dominance, may simply have less selective value to preserve in a lineage that does not practice single-follicle selection, rather than reflecting a fundamental absence of CART’s inhibitory capacity at the molecular level in porcine granulosa cells specifically [51]. This generates a testable, falsifiable prediction that follows directly from combining Li et al.’s (2018) functional data with Ahn et al.’s (2014) evolutionary data: CART’s inhibitory effect on FSH-stimulated steroidogenesis should be measurably stronger, across a systematically sampled panel of species, in monotocous and oligotocous species than in strongly polytocous ones, tracking reproductive strategy rather than phylogenetic distance from cattle [51,64]. The hen data complicate this prediction somewhat, since domestic fowl are neither monotocous in the mammalian sense nor comparably polyovulatory to pigs, and sit outside the framework Ahn et al.’s (2014) mammal-only comparison was built to address, a reminder that the monotocous-polytocous framing, however useful for interpreting the pig result specifically, does not extend cleanly across the full taxonomic range this review has covered [64].

The four sources of human evidence surveyed in Chapter 8 were generated in different patient cohorts, using different measurement panels, and were not designed to be read against one another, which makes any synthesis here necessarily provisional. Ma et al. (2016) reported a direct positive correlation between BMI and *CARTPT* expression in human granulosa cells and follicular fluid [12]. Jafarpour et al. (2021), pooling fourteen studies, found no statistically significant relationship between follicular fluid leptin and IVF outcome [58]. Xu et al. (2019) reported an insulin-driven, leptin-independent mechanism suppressing *FSHR* expression in obese women undergoing IVF [40]. Voros et al. (2024) reported, in a substantially larger cohort, that follicular fluid CART correlates negatively with oocyte yield and maturity [38], the first human data linking CART to an outcome beyond BMI, and directionally consistent with Ma et al.’s premise that higher CART marks impaired follicular function.

One specific point of tension among these three studies is worth surfacing rather than passing over. Xu et al. (2019) explicitly report that in their cohort, insulin and glucose correlated with BMI in follicular fluid, but follicular fluid leptin did not, a finding that, if it generalizes, would be difficult to reconcile with the premise underlying Ma et al.’s (2016) work and much of the literature Jafarpour et al. (2021) synthesized, namely that BMI reliably predicts follicular fluid leptin concentration in women undergoing IVF [12,40,58]. A plausible explanation for this apparent contradiction, rather than a simple methodological discrepancy between the two study cohorts, comes from the broader PCOS and obesity literature on metabolic phenotype heterogeneity. Al-Jefout et al. (2017), studying insulin resistance across 213 infertile women with and without PCOS, found that insulin resistance was significantly associated with PCOS only among women with central obesity specifically, and not among those in the normal-weight category, with clear stratification across PCOS phenotypes in the degree of hyperinsulinemia observed [65]. If leptin elevation and insulin resistance track different, only partially overlapping subsets of obese infertile women, with leptin rising roughly in proportion to overall adiposity and insulin resistance clustering more specifically with central, visceral fat distribution and particular PCOS phenotypes, as Al-Jefout et al.’s (2017) phenotype-stratified data suggest, then Ma et al.’s (2016) cohort and Xu et al.’s (2019) cohort could easily have differed in their proportion of centrally obese, insulin-resistant patients versus more generally overweight, leptin-elevated patients without either study’s BMI-based inclusion criteria capturing that distinction, producing exactly the kind of divergent primary correlate each study reports [12,40,65].

This reframes the discrepancy between Ma et al. (2016) and Xu et al. (2019) as potentially reflecting real, clinically meaningful patient heterogeneity rather than a simple failure of one study or the other to replicate a single underlying relationship [12,40]. It would imply that obesity-related IVF impairment is not a single uniform phenotype but may run predominantly through the leptin-CART axis Ma et al. (2016) describe in some patients, plausibly those with elevated BMI but less central, metabolically active adiposity, and predominantly through the insulin-GSK3β-FSHR axis Xu et al. (2019) describe in others, plausibly those with the central obesity and insulin resistance phenotype Al-Jefout et al. (2017) characterize [12,40,65]. No study has yet been designed to test this directly by stratifying patients on both leptin and insulin resistance status simultaneously and measuring *CARTPT*, FSHR expression, and IVF outcome across the resulting subgroups, and doing so is one of the more concrete, well-motivated next steps this review’s cross-study reading points toward, developed further in Chapter 10.

## 12. Conclusions

This review has traced *CARTPT* from an incidental finding in a bovine oocyte cDNA library to a candidate local regulator of follicle selection with a plausible, though still incomplete, link to human obesity-related infertility. The evidence is strongest at its origin: in cattle, CART’s suppression of FSH-stimulated cAMP, ERK1/2 and Akt signaling, and aromatase expression in granulosa cells rests on a coherent, mutually reinforcing body of work spanning expression mapping, receptor binding characterization, and interventional confirmation. This mechanism extends, with modification, to buffalo through a related but biochemically distinct pathway, and finds a plausible upstream trigger in leptin, consistent with CART’s original identity as a hypothalamic satiety factor.

The evidence weakens, in ways this review has tried to state precisely rather than gloss over, as it moves outward from this core. The porcine data suggest CART’s role may be tied to reproductive strategy rather than universally conserved. The CART receptor remains molecularly unidentified in every species studied. And the direct human CARTPT evidence rests on two correlational studies set against a much larger but internally inconsistent literature on the upstream leptin signal.

None of this diminishes the value of *CARTPT* as a subject of continued investigation; if anything, the specificity of the mechanism, in contrast to the broader and noisier leptin literature, is what makes it worth pursuing further. But it does mean that *CARTPT* should be understood, at this stage, as a well-characterized preclinical mechanism with biological plausibility in humans, not as a validated clinical marker or a fully resolved account of how metabolic status shapes oocyte quality. The path from here to clinical relevance runs through the specific, identified gaps in Chapter 10: receptor identification, loss-of-function confirmation, and integrated human phenotyping, each of which is answerable with existing experimental tools, applied to a question the field has not yet asked in quite this combination.

## Figures and Tables

**Table 1:** Cross-species evidence for CARTPT-mediated regulation of FSH signaling and follicular fate.

Species	Model/System	Key Finding	Proposed Mechanism	Reference(s)
Cattle	Granulosa cells, in vivo follicular wave	*CARTPT* elevated in subordinate/atretic follicles before morphological atresia; suppresses FSH-stimulated cAMP, ERK1/2, Akt, and *CYP19A1*/estradiol	cAMP-PKA suppression; accelerated ERK1/2-Akt inactivation via MAPK phosphatases	[5,6,7,8,10]
Cattle	Granulosa cells, follicular wave	FSH reciprocally suppresses *CARTPT* expression	WNT/β-catenin-dependent transcriptional suppression	[39]
Water buffalo	Granulosa cells, atretic vs. healthy follicles	*CARTPT*/*BAX* elevated in atretic follicles; CART reduces viability and estradiol, increases apoptosis	AKT/GSK3β/β-catenin suppression, downstream *CYP19A1* loss	[35]
Pig	Granulosa cells, follicles stratified by size	CART effect on FSH-stimulated estradiol non-significant	Proposed: CART not a major local regulator in a polyovulatory species	[51]
Domestic hen	Ovarian follicles, thecal/granulosa layers	*CARTPT* localizes predominantly to theca; expression differs by follicle size class	Not functionally tested	[34]
Mouse	Granulosa cells, diet-induced obesity model	*Cartpt* induced by high-fat diet and by leptin administration; not detected under baseline conditions	Leptin-JAK2-STAT3-induced *Cartpt*; suppresses cAMP, *Cyp19a1*, ovulation	[12]
Human	Granulosa cells/follicular fluid, IVF patients	*CARTPT* mRNA and CART peptide correlate positively with BMI	Proposed leptin-CART induction, extrapolated from the mouse model	[12]
Human	Follicular fluid, 94 IVF patients, four stimulation protocols	CART negatively correlated with follicle count >12 mm, oocyte yield, and oocyte maturity	Consistent with inhibitory CART-FSH mechanism; protocol- and genotype-dependent	[38]

Table 1 legend: FSH, follicle-stimulating hormone; PKA, protein kinase A; ERK1/2, extracellular signal-regulated kinase 1/2; Akt, protein kinase B; CYP19A1, aromatase; BAX, BCL2-associated X protein; GSK3β, glycogen synthase kinase 3 beta; BMI, body mass index. Findings are ordered from the most extensively characterized (cattle) to the least (human), reflecting the depth rather than the chronology of the underlying evidence. Human findings are limited to two correlational cohort studies and should not be read as clinically validated.

**Table 2:** Human studies relating follicular fluid leptin or CART to IVF outcomes.

Study Type	Cohort/Design	Measure	Key Finding
Prospective cohort, [55]	35 normogonadotrophic women, IVF	Follicular fluid leptin vs. fertilization	Leptin > 20.25 ng/mL associated with 85.7% fertilization rate vs. 16.7% below threshold
Prospective cohort, [56]	Women stratified by ovarian reserve (AMH), IVF	Follicular fluid leptin vs. oocyte maturity	Leptin predicted oocyte maturity (AUC 0.829); cut-off 16 ng/mL
Cohort, individual follicles, [57]	30 women, 84 follicles, IVF	Follicular fluid leptin and soluble leptin receptor vs. oocyte quality	No correlation with oocyte quality, fertilization, or embryo grade
Systematic review and meta-analysis, [58]	14 pooled studies, IVF/ICSI	Serum and follicular fluid leptin vs. pregnancy outcome	No statistically significant difference in leptin between pregnant and non-pregnant women
Case–control mechanistic study, [40]	Overweight/obese vs. normal-weight women, IVF	*FSHR* expression; insulin, glucose, and leptin in follicular fluid	*FSHR* reduced in higher-BMI groups; correlated with insulin/glucose, not with leptin; mediated via AKT2/GSK3β
Correlational cohort, [12]	Women undergoing IVF	*CARTPT* mRNA and CART peptide vs. BMI	Positive correlation between BMI and both *CARTPT* mRNA and follicular fluid CART peptide
Prospective cohort, [38]	94 women, four stimulation protocols, IVF	Follicular fluid CART vs. follicle count, oocyte yield, oocyte maturity	Negative correlation with all three; FSHR Ser680Ser genotype associated with higher CART

Table 2 legend: AMH, anti-Müllerian hormone; AUC, area under the receiver operating characteristic curve; FSHR, follicle-stimulating hormone receptor; BMI, body mass index.

**Table 3:** Key knowledge gaps in the CARTPT–FSH pathway and proposed experiments to resolve them.

Knowledge Gap	Current State of Evidence	Proposed Experiment	Relevant Chapter(s)
Ovarian CART receptor identity remains unresolved	GPR160 proposed then excluded by direct binding studies; GPR162 since identified as a genuine CART receptor in pancreatic islet cells [19] but untested in the ovary; granulosa cell binding sites characterized but molecularly unidentified	GPR162 expression profiling and radioligand binding in granulosa cells, paired with knockdown to confirm functional relevance	3, 5
Causality vs. correlation in follicle selection	Only gain-of-function data available (exogenous CART reproduces subordinate-follicle phenotype); no loss-of-function evidence	Neutralize or knock down endogenous CART within an intact, developing follicular wave and assess whether subordinate follicles are rescued	6
Cross-species generalizability of the monotocous/polytocous hypothesis	Functional data exist in only five species (cattle, buffalo, pig, hen, mouse), sampled opportunistically rather than systematically	Test CART’s inhibitory effect on FSH-stimulated steroidogenesis across a panel of species deliberately spanning the monotocous–polytocous axis	4, 9
Proposed convergence of cAMP-PKA and AKT/GSK3β mechanisms	Biochemically plausible (PKA and Akt both inhibit GSK3β via Ser9) but Axin scaffolding may insulate the Wnt-relevant GSK3β pool from this cross-talk	Measure Axin–GSK3β complex composition alongside cAMP and Akt signaling in the same CART-treated granulosa cells, in both cattle and buffalo	9
Integrated human phenotyping across obesity-related pathways	CARTPT, follicular fluid leptin, and insulin resistance status have never been measured together in the same IVF patients	Prospective cohort stratifying obese IVF patients by both leptin and insulin-resistance status, measuring CARTPT, FSHR expression, and IVF outcome across subgroups	8, 9

Table 3 legend: FSHR, follicle-stimulating hormone receptor; GSK3β, glycogen synthase kinase 3 beta. Chapter numbers refer to this manuscript’s structure. Proposed experiments are ordered to match the sequence in which the corresponding gaps are discussed in Chapter 10.

## Data Availability

No new data were created or analyzed in this study. Data sharing is not applicable to this article.
